# Genomic approaches for the elucidation of genes and gene networks underlying cardiovascular traits

**DOI:** 10.1007/s12551-018-0435-2

**Published:** 2018-06-22

**Authors:** M. E. Adriaens, C. R. Bezzina

**Affiliations:** 1 Maastricht Centre for Systems Biology, Maastricht University, Universiteitssingel 60, 6229 ER Maastricht, The Netherlands; 2 Department of Clinical and Experimental Cardiology, Heart Center, Academic Medical Center, University of Amsterdam, Meibergdreef 9, 1105 AZ Amsterdam, The Netherlands

**Keywords:** GWAS, Cardiovascular, RNA-seq, Allele-specific expression, Systems genetics

## Abstract

Genome-wide association studies have shed light on the association between natural genetic variation and cardiovascular traits. However, linking a cardiovascular trait associated locus to a candidate gene or set of candidate genes for prioritization for follow-up mechanistic studies is all but straightforward. Genomic technologies based on next-generation sequencing technology nowadays offer multiple opportunities to dissect gene regulatory networks underlying genetic cardiovascular trait associations, thereby aiding in the identification of candidate genes at unprecedented scale. RNA sequencing in particular becomes a powerful tool when combined with genotyping to identify loci that modulate transcript abundance, known as expression quantitative trait loci (eQTL), or loci modulating transcript splicing known as splicing quantitative trait loci (sQTL). Additionally, the allele-specific resolution of RNA-sequencing technology enables estimation of allelic imbalance, a state where the two alleles of a gene are expressed at a ratio differing from the expected 1:1 ratio. When multiple high-throughput approaches are combined with deep phenotyping in a single study, a comprehensive elucidation of the relationship between genotype and phenotype comes into view, an approach known as systems genetics. In this review, we cover key applications of systems genetics in the broad cardiovascular field.

## Introduction

Over the last decade, genome-wide association (GWA) studies have shed light on the association between natural genetic variation and cardiovascular traits. GWA studies have been conducted, largely in individuals of European descent, for sudden cardiac death (Arking et al. 2011; Bezzina et al. 2010); atrial fibrillation (Benjamin et al. 1994; Christophersen et al. 2017b; Ellinor et al. 2010, 2012; Gudbjartsson et al. 2007, 2009; Lee et al. 2017; Low et al. 2017); resting heart rate (Cho et al. 2009; den Hoed et al. 2013; Deo et al. 2013; Eijgelsheim et al. 2010; Eppinga et al. 2016; Holm et al. 2010; Marroni et al. 2009; Mezzavilla et al. 2014; Nagy et al. 2017; Noordam et al. 2017); heart rate variability (Kerr et al. 2017; Nolte et al. 2017); PR interval (Butler et al. 2012; Chambers et al. 2010; Christophersen et al. 2017a; Hong et al. 2014; Pfeufer et al. 2010; Smith et al. 2011; Verweij et al. 2014); QRS duration (Evans et al. 2016; Floyd et al. 2018; Holm et al. 2010; Hong et al. 2014; Ritchie et al. 2013; Sotoodehnia et al. 2010; van der Harst et al. 2016); ST and JT duration (Floyd et al. 2018; Verweij et al. 2016); and QT interval (Arking et al. 2006, 2014; Chambers et al. 2010; Floyd et al. 2018; Holm et al. 2010; Kim et al. 2012; Marroni et al. 2009; Newton-Cheh et al. 2009; Nolte et al. 2009; Pfeufer et al. 2009; Sano et al. 2014; Smith et al. 2012). However, of the hundreds of loci that have been identified within these studies, most are intergenic and do not directly affect protein sequence or function, instead impacting on regulation of gene expression and splicing (Heinig et al. 2017; Koopmann et al. 2014), thereby requiring the generation of additional gene expression data in relevant tissues. Secondly, taking into account the non-linear nature of regulatory chromatin interactions, a locus can interact with any one or multiple genes in *cis*, as shown previously for the *SCN5A* locus which houses several important sodium channel genes (van den Boogaard et al. 2014). Thirdly, the underlying statistical modeling foundations of GWA studies seldom take epistatic effects into account, due to the low power associated with formal testing of such gene-gene interactions (Ma et al. 2015). Hence, linking a cardiovascular trait associated locus to a candidate gene or set of candidate genes for prioritization for follow-up mechanistic studies is all but straightforward.

Genomic technologies based on next-generation sequencing technology nowadays offer multiple opportunities to dissect gene regulatory networks underlying genetic cardiovascular trait associations, thereby aiding in the identification of candidate genes at unprecedented scale. Genome-wide chromosome conformation capture techniques (3C, 4C, 5C, and Hi-C) have demonstrated that chromatin interactions in human cells occur predominantly within domains with an average size of 880 Kb, known as topologically associated domains (TADs). These TADs are shared across a variety of cell types and are suggested to align with replication sites (Dixon et al. 2016) and causal genes underlying a GWAS locus are likely to reside in the same TAD as the haplotype carrying the associated SNP. Meanwhile, chromatin immunoprecipitation (ChIP) sequencing has opened the path to studying a plethora of histone modifications, which when combined can pinpoint specific regulatory regions within TADs, such as enhancers, promoters, silencers, and insulators, at an unprecedented resolution (ENCODE-Project-Consortium 2012; Zhou et al. 2011). Additional high-resolution chromosome conformation capture techniques subsequently enables revealing of the interactions between regulatory regions, for example showing the gene promoters that a specific enhancer associates with as in the case of the *SCN10A* enhancer interacting with the *SCN5A* locus (van den Boogaard et al. 2014). Finally, RNA sequencing (RNA-seq) technology can reveal the consequence of these chromatin interactions on the expression and splicing of genes.

RNA-seq becomes a particularly powerful tool when combined with genotyping to identify loci that modulate transcript abundance, known as expression quantitative trait loci (eQTL), or loci modulating transcript splicing known as splicing quantitative trait loci (sQTL). Additionally, the allele-specific resolution of RNA-seq technology enables estimation of allelic imbalance, a state where the two alleles of a gene are expressed at a ratio differing from the expected 1:1 ratio. Key mechanisms underlying allelic imbalance include the presence of truncating variants leading to nonsense-mediated decay of one of the alleles (Castel et al. 2015), genetic regulation via *cis* eQTLs and sQTLs, as well as epigenetic modifications through imprinting and interactions with the environment (Castel et al. 2015; Gutierrez-Arcelus et al. 2013; Gutierrez-Arcelus et al. 2015). The genetic effects can be calculated using standardized procedures (Shabalin 2012), after which the epigenetic effect can be reliably established through statistical inference (Knowles et al. 2017).

Genomic datasets like the ones described above are increasingly available in the public domain for data reuse and integration. A prime example, the ambitious database of GTEx (GTEx-Consortium et al. 2017) offers data on genetic regulation of expression (eQTL) and splicing (sQTL) in 44 human tissues, including cardiac tissues, at respectable sample sizes. Meanwhile, the current Ensembl database offers integrated information of regulatory regions, such as enhancers and promoters, in a plethora of different cell types and tissues (Zerbino et al. 2018), including cardiac tissues, based in part on the results from the ENCODE project (ENCODE-Project-Consortium 2012). One step further, the integrative web-based platform FUMA (Watanabe et al. 2017) uses information from multiple biological resources to facilitate functional annotation of GWA study results and gene prioritization by accommodating positional, eQTL, and chromatin interaction mappings, and provides gene-based, pathway, and tissue enrichment results. FUMA has recently been successfully applied in GWA studies on insomnia (Hammerschlag et al. 2017) and dementia (Sniekers et al. 2017). It should be noted that all these resources are updated on a regular basis, hence using the most recent online version should be common practice in their application.

The above would suggest that integrative interpretation is mainly restricted by the available computational expertise and resources. However, an important limitation of public resources is that the majority of data is derived from tissues of non-diseased origin. Additionally, tissues are composed of many different cell types, with each comprising a specific gene expression profile and set of gene regulatory networks (GTEx-Consortium et al. 2017). In assessing differences between experimental conditions, this may result in spurious findings due to variations in cell type composition, or conversely, the inability to identify differences due to underlying mechanisms being cell type specific. Finally, biological differences between different study populations, for example with respect to genetic and epigenetic background and phenotypic characteristics, as well as technical differences, such as experimental conditions and sample collection and handling, are often unknown, leading to residual latent confounding that comes at the cost of statistical power. Ideally, multiple high-throughput approaches are combined with deep phenotyping in a single study, integrating genetic variants with molecular phenotypes to more comprehensively elucidate the relationship between genotype and phenotype, an approach known as systems genetics (Civelek and Lusis 2014; Moreno-Moral et al. 2017). Here, we cover several key applications of systems genetics in the broad cardiovascular field.

## The principles underlying systems genetics approaches

The starting point of systems genetics approaches remains quantitative trait locus (QTL) analysis, identifying genetic variants modulating a specific quantitative trait. By subsequently overlapping the QTL with eQTL and sQTL data, either generated within the study or retrieved from public repositories (GTEx-Consortium 2013; GTEx-Consortium et al. 2017), candidate genes that are under genetic control with regard to overall expression or splicing can be identified. In case such genes are found, their respective regulatory networks should be further explored by integrating additional information. This may consist of predictive information, such as protein-protein interactions (Szklarczyk et al. 2017), shared function via Gene Ontology (Alexa et al. 2006), shared transcription factor binding sites (Roider et al. 2009) shared interacting regulatory molecules such as miRNAs (Backes et al. 2016), or a combination of these. Better yet, regulatory interactions can be implied by using molecular data generated within the study. The most common approach is using the genome-wide transcript abundance to construct co-expression networks (Langfelder and Horvath 2008). Alternatively, one could look at shared epigenetic markings, such as histone modifications (Rintisch et al. 2014) or DNA methylation (Gutierrez-Arcelus et al. 2013, 2015; Lappalainen et al. 2013), to infer regulatory relationships between genes.

Historically, the statistical framework underlying QTL analyses has been based on standard linear regression approaches, where the individual relation between each variant and a trait is tested independently. Besides feeding into a substantial multiple testing problem, which can be partly compensated by appropriate methods (Johnson et al. 2010), it ignores the linkage disequilibrium (LD) between SNPs (i.e., *cis* inter-dependence), as well as the presence of epistatic effects (i.e., *trans* inter-dependence). Bayesian approaches offer a means for true multi-variate statistical analysis that considers these aspects. Bayesian approaches for QTL analysis have been successfully applied to genetic association studies in animal models (Bottolo et al. 2011a, b). While statistically more robust and—by taking into account interdependence of genetic variants—more biologically sound than standard one-by-one linear regression approaches, a clear limitation is the required computational time. Hence, while it has been successfully applied in animal models (Bottolo et al. 2011b; Heinig et al. 2010; Moreno-Moral et al. 2013; Petretto et al. 2008, 2010; Rintisch et al. 2014), the genetic heterogeneity of human populations hampers the application of Bayesian methods.

Recent advances however have enabled the development of computationally accelerated and parallelized algorithms (Lewin et al. 2015), enabling the application of Bayesian methods to human genetic studies as well. Das et al. (2015) developed eQTeL, a Bayesian algorithm that infers *cis* regulatory polymorphisms underlying gene expression variability by integrating (i) genotype and gene-expression variance across individuals; (ii) epigenetic data, (iii) DNAse I hypersensitivity variance of SNPs and promoters, and (iv) expression variance of genes across multiple cell types; in addition to (v) LD blocks and (vi) imputed haplotypes inferred from the 1000 Genomes Project (Das et al. 2015). Importantly, eQTeL is scalable to large datasets.

## Applications of systems genetics in the study of cardiovascular traits

Concerning systems genetics studies of complex traits in the cardiovascular domain, the HXB/BXH panel of recombinant inbred (RI) rat strains (Pravenec et al. 1989) remains a leading model (Heinig et al. 2010; Hubner et al. 2005; Moreno-Moral and Petretto 2016; Printz et al. 2003). The panel was generated by reciprocal crossing of the Brown Norway (BN) rat and the spontaneously hypertensive rat (SHR). Briefly, the construction of an RI panel involves mating two inbred genetically distant progenitor strains to produce F2 hybrids that carry a unique combination of maternal and paternal loci. Subsequently, individual homozygous RI strains are generated by inbreeding of randomly chosen pairs of F2 animals and brother-sister mating for more than 20 generations (Silver 1995). This results in a panel that offers a controllable, renewable resource that combines genetic identity within strains with genetic diversity across strains. A comprehensive overview of the HXB/BXH RI panel and its applications can be found in Moreno-Moral and Petretto (2016).

RI panels are particularly powerful for genetic studies, as the study of genetically identical biological replicates optimizes estimation of trait heritability by reducing environmental variance, while the constant genetic background within each RI strain allows for the accumulation of genetic, omics, and phenotypic data over time. For the HXB/BXH RI rat panel, genotyping of the RI strains has led to the identification of 1384 strain distribution patterns (SDPs) of single nucleotide polymorphisms (SNPs) for use in genetic mapping (Star-Consortium et al. 2008). Full genomic sequencing data is available for both parental strains (Atanur et al. 2010; Simonis et al. 2012), in addition to RNA-seq-based transcript abundance data for multiple tissues across the panel (Johnson et al. 2014) and a plethora of cardiovascular physiological traits such as blood pressure and cardiac mass (Pravenec et al. 1995). The HXB/BXH RI panel therefore lends itself exceptionally well to the study of complex cardiovascular traits.

One of the first systems genetics studies performed within the HXB/BXH panel was published in 2008 by Petretto et al. (2008). In that study on the genetics underlying left ventricular mass (LVM), a combination of QTL and transcriptomics analysis pinpointed a single gene, *Ogn*, as a major candidate regulator. Two years later, Heinig et al. (2010) through combined analyses of gene networks and genetic variation identified an interferon regulatory factor 7 (*Irf7*)-driven inflammatory network enriched for viral response genes. The expression of genes in this network was shown to be regulated in multiple tissues by a single locus on chromosome 15. Subsequent analysis of the orthologous region and orthologous genes in human demonstrated association with type 1 diabetes for both, implicating the implicate *Irf7* network genes and their regulatory locus in the pathogenesis of this complex cardiometabolic disease. Petretto et al. (2010) extended this approach by hunting for eQTL hotspots, loci that modulate gene expression across a wide variety of tissues, using a multi-variate Bayesian approach (Bottolo et al. 2011b; Lewin et al. 2015). They demonstrated common genetic regulation of gene expression across four tissues for approximately 27% of all expressed transcripts, providing a more than fivefold increase in eQTL detection rate compared to single tissue analyses. Further extending this integrative approach, Morrissey et al. (2011) combined a quantitative trait transcript (QTT) analysis, i.e. genes that correlate with clinically relevant cardiometabolic traits such as systolic blood pressure and blood glucose, with *cis* eQTLs and cardiometabolic trait QTLs. They proposed that these co-localizing correlated *cis* eQTLs (c3-eQTLs) are highly attractive for prioritization and identified multiple candidate genes as strong positional candidates for the investigated cardiometabolic traits. Lastly, Moreno-Moral et al. (2013) combined left ventricular myocardium co-expression networks with histological and histomorphometric data of the heart and coronary vasculature, to provide a large catalog of gene co-expression networks in the heart that are significantly associated with quantitative variation in left ventricular hypertrophy, microvascular remodeling, and fibrosis-related traits. Additionally, they demonstrated the relevance of co-expression networks identified in rat for human heart disease, by showing that many of these networks were shown to be significantly conserved in left ventricular myocardium of human idiopathic and ischemic cardiomyopathy patients.

More recently, the HXB/BXH panel was used to study the interplay between genetic variation and the presence of specific histone modifications (Rintisch et al. 2014). These epigenetic marks known to be important in the modulation of biological processes, including regulation of gene expression through control of chromatin structure. The study by Rintisch et al. (2014) focused on the histone methylation marks H3K4me3 (associated with active gene promoters), H3K27me3 (associated with silenced gene promoters, and more specifically Polycomb-repressed regions), H3K4me1 (associated with gene promoter and enhancer regions), and H4K20me1 (associated with recently transcribed genomic regions). Additionally, genome-wide genetic variation and left ventricular transcript abundance were assessed for the entire panel. The results of this study showed that genetic variants associated with differential histone modifications, referred to as histoneQTLs, are a predictor of gene expression, with the most prominent QTL identified influencing H3K4me3 levels at 899 gene promoters in the heart, proving the important role of histone modifications in genotype-phenotype relationships.

One of our own recent efforts (*under review*) pertaining to the HXB/BXH RI rat panel involved mapping genetic loci impacting on heart rate and ECG indices of cardiac conduction. QTL analysis on ECG parameters identified two QTL for PR-interval, respectively, on chromosomes 10 and 17. By further intersection of this data with cardiac eQTL data, we identified novel candidate genes associated with cardiac conduction. Subsequently expanding these findings by constructing co-expression networks enabled us to identify multiple gene networks associated with cardiac conduction, shown to be enriched for cardiac related processes through Gene Ontology enrichment analysis.

However powerful, translating genetic findings from animal models to human without functional validation is by nature limited through inference (Heinig et al. 2010; Petretto et al. 2008). Several large studies in human have focused on investigating gene expression regulation in the heart. Koopmann et al. (2014) reported on an eQTL analysis of human left ventricular tissue of 129 non-diseased donors, creating the first comprehensive eQTL map of human heart. The study identified 771 eQTLs regulating 429 unique genes, while overlaying these eQTLs with cardiovascular trait, GWAS loci identified novel candidates for follow-up studies to further elucidate the functional and transcriptional impact of these loci. In the same year, Lin et al. (2014) reported on the characterization of gene expression and genetic variation in 53 human left atrial and 52 right atrial tissue samples collected from the Myocardial Applied Genomics Network (MAGNet) repository. Next to identifying a distinct transcriptional profile between the right and left atrium and extensive *cis* associations between atrial transcripts and common genetic variants, their approach identified a causative gene for AF, *MYOZ1*.

More recently, the study from Heinig et al. (2017) reports on the first in-depth analysis of the dilated cardiomyopathy (DCM) transcriptome. Myocardial ischemia as well as toxic, metabolic immunologic factors can lead to the DCM phenotype. Moreover, genetic susceptibility plays an important role, with at least 23% of DCM cases being familial and more than 50 genes linked to inherited DCM. By performing RNA-sequencing analysis in left ventricular biopsies or 97 patients with DCM and 108 non-diseased controls, they revealed extensive differences of gene expression and splicing between patients and controls, in addition to a widespread effect of genetic variation on the regulation of transcription, isoform usage, and allele-specific expression. Strikingly, these differences did not just pertain to known DCM genes but extended to a wide range of novel candidates. Overlaying their findings with genome-wide association, SNPs identified 60 functional candidate genes for cardiovascular traits. This represented 20% of all published heart genome-wide association loci at the time, a much larger set of candidates than previously identified (Koopmann et al. 2014). Finally, it was shown that eQTL variants are also enriched for dilated cardiomyopathy genome-wide association signals in two independent cohorts. Their conclusion that genetic regulation of RNA transcription, splicing, and allele-specific expression are important determinants for the DCM phenotype likely extends to many other cardiovascular traits.

Taken together, these studies suggest that combining genome-wide genotyping with transcript abundance data forms the powerful foundations of systems genetic approaches.

## Conclusions and prospects

Throughout the genetic studies discussed within this review, the loci and gene networks associated with the complex cardiovascular traits provide a starting point for future studies with the potential of identifying novel underlying mechanisms. The take-home message is that the main conclusion of all these studies could not have been reached without the applied integrative approaches: combining multiple measures of transcriptional control in a presence of a complex genetic background with multiple multi-variate analyses techniques.

Lastly, early detection combining molecular markers and clinical parameters is pivotal for preventive medicine in cardiology, but due to the heterogeneity of many diseases, this has proven to be exceptionally difficult. In the recent paper by Heinig et al. (2017), by exploiting the allele specificity of RNA-sequencing through allele-specific expression (ASE) analysis, it was shown that in DCM hearts, dysregulation occurs of only one of the two alleles for known DCM progressing genes. Allelic imbalance was for example observed for *TTN*, a gene for which many truncating variants have been identified that can lead to nonsense-mediated decay (Schafer et al. 2017). Strikingly, there was no clear pattern emerging, in line with earlier observations that were made in a subset of DCM samples (Roberts et al. 2015). For the DCM phenotype, this could indicate that imbalance shifts towards disease-contributing alleles during disease progression. Looking beyond *TTN*, both the extent of the allelic imbalance and the number of known DCM genes affected varied greatly from patient to patient, yet all differential allelic imbalanced genes combined showed enrichment for DCM-related processes, as well as differential splicing and miRNA interference. This suggests a complex, heterogeneous genetic dosage effect across individuals, that in many cases will not be identifiable by standard GWAS, eQTL, and sQTL analyses. Additionally, this could suggest that such allelic imbalances are related to disease stage and may accumulate over time by interplay between genetic and epigenetic mechanisms, enabling early detection. Contrasting regular gene expression analysis, allelic imbalance can be reliably measured within a single individual, and several recent developments enable a statistically robust analysis (Knowles et al. 2017; Leon-Novelo et al. 2014). Taken together, ASE analysis has the potential to enable a full personalized, quantitative genetic typing for clinical prognosis that goes beyond established qualitative assays detecting solely the presence of genetic variants, as well as enable studying the interaction between genetic and epigenetic regulation in the establishment of the imbalance for complex cardiovascular traits.
